# Supplementary material for: The Role of Age, Neutrophil Infiltration and Antibiotics Timing in the Severity of Streptococcus pneumoniae Pneumonia. Insights from a Multi-Level Mathematical Model Approach
Source: Int J Mol Sci. 2020 Nov 10;21(22):8428. doi: 10.3390/ijms21228428 (PMC7696447; doi:10.3390/ijms21228428)
Supplement: Supplementary file 1 [file ijms-21-08428-s001.pdf]

The role of age, neutrophil infiltration and antibiotics timing in the severity of *Streptococcus pneumoniae* pneumonia. Insights from a multi-level mathematical model approach

Guido Santos[1,2], Julio Vera[1]

http://orcid.org/0000-0002-4231-5946

https://orcid.org/0000-0002-3076-5122

1. Laboratory of Systems Tumor Immunology, Department of Dermatology, Universitätsklinikum Erlangen and Faculty of Medicine, Friedrich-Alexander University Erlangen-Nürnberg, Erlangen, Germany.
2. Universidad de La Laguna, Departamento de Bioquímica, Microbiología, Biología Celular y Genética. San Cristóbal de La Laguna, España.

Supplementary material

Young: 11 bacteria, 12 h.

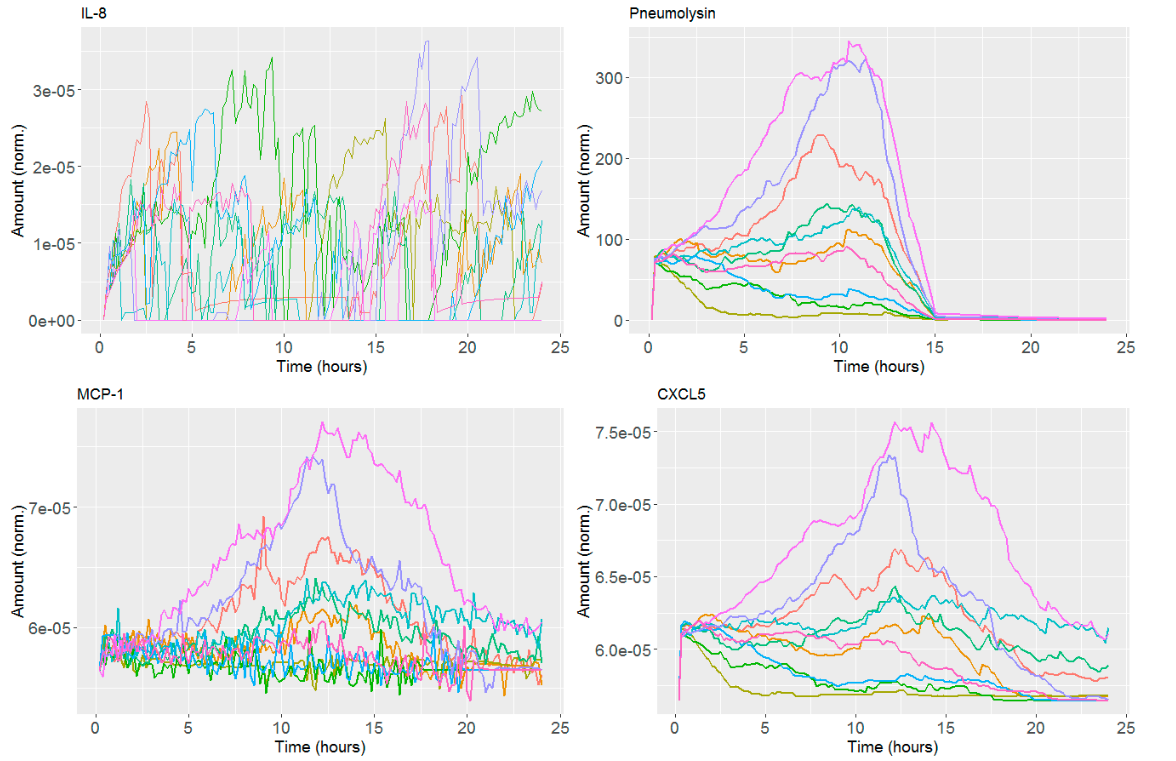

Young: 11 bacteria, 20 h.

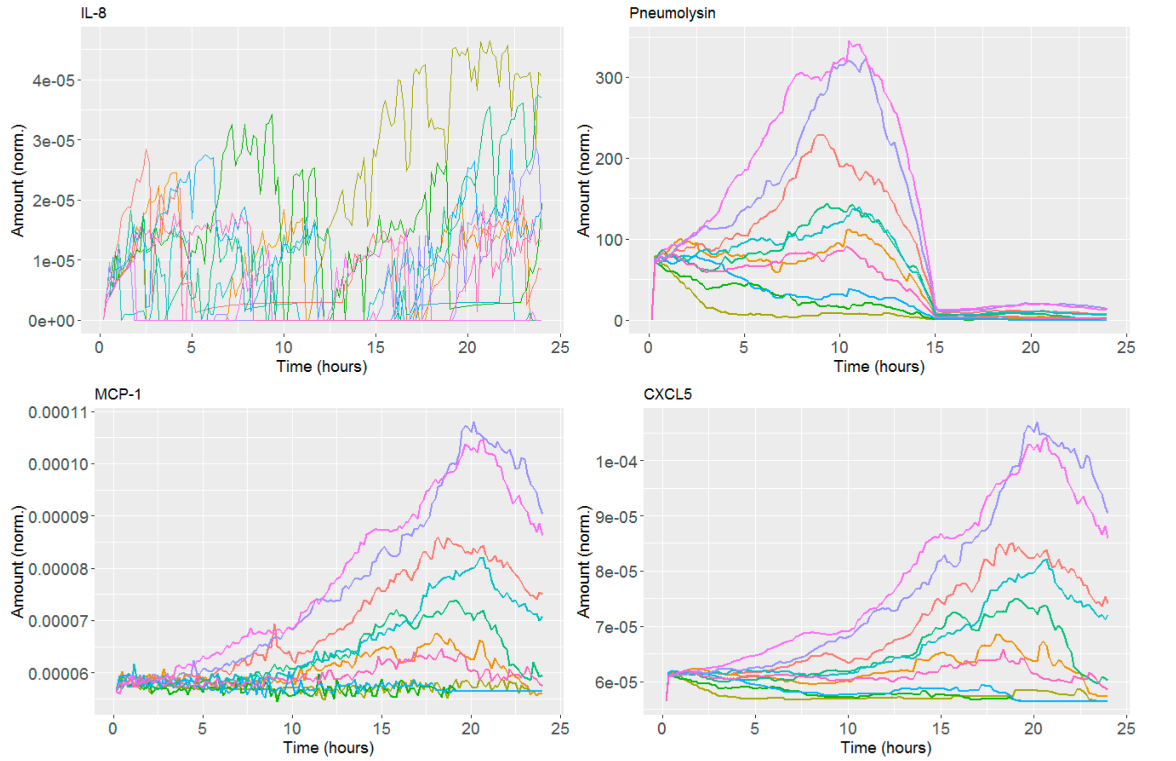

Aged: 11 bacteria, 12 h.

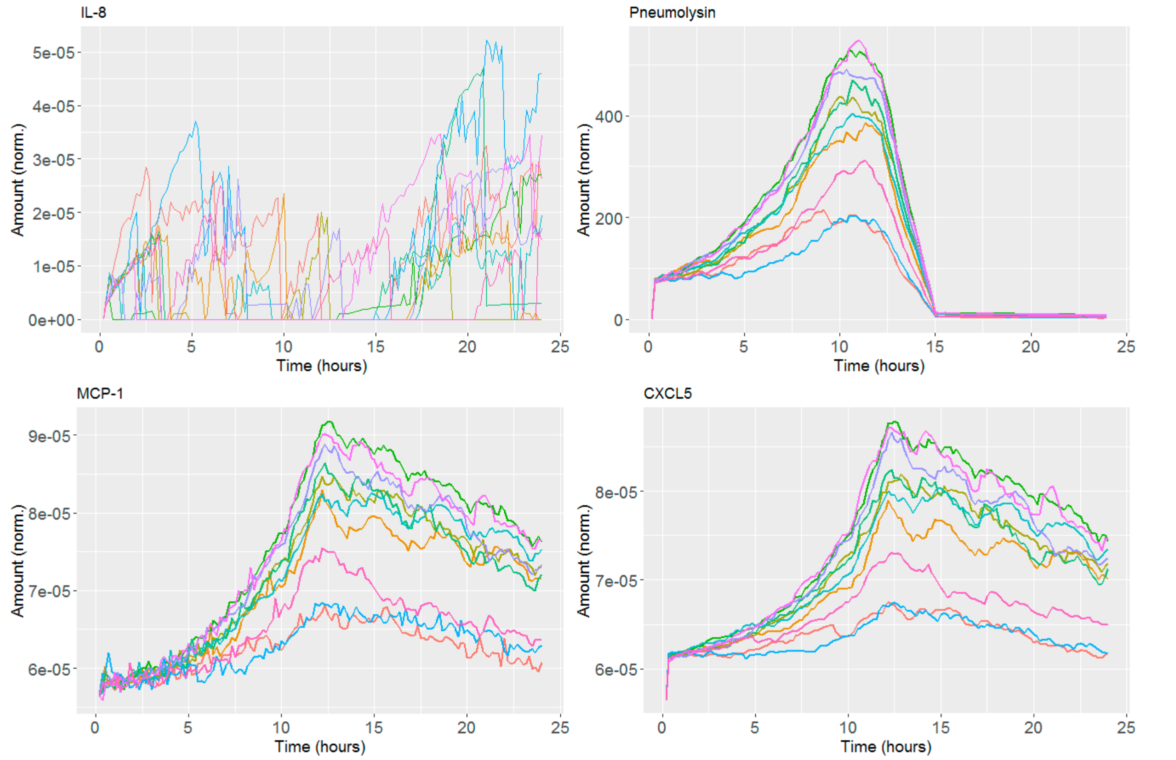

Aged: 11 bacteria, 20 h.

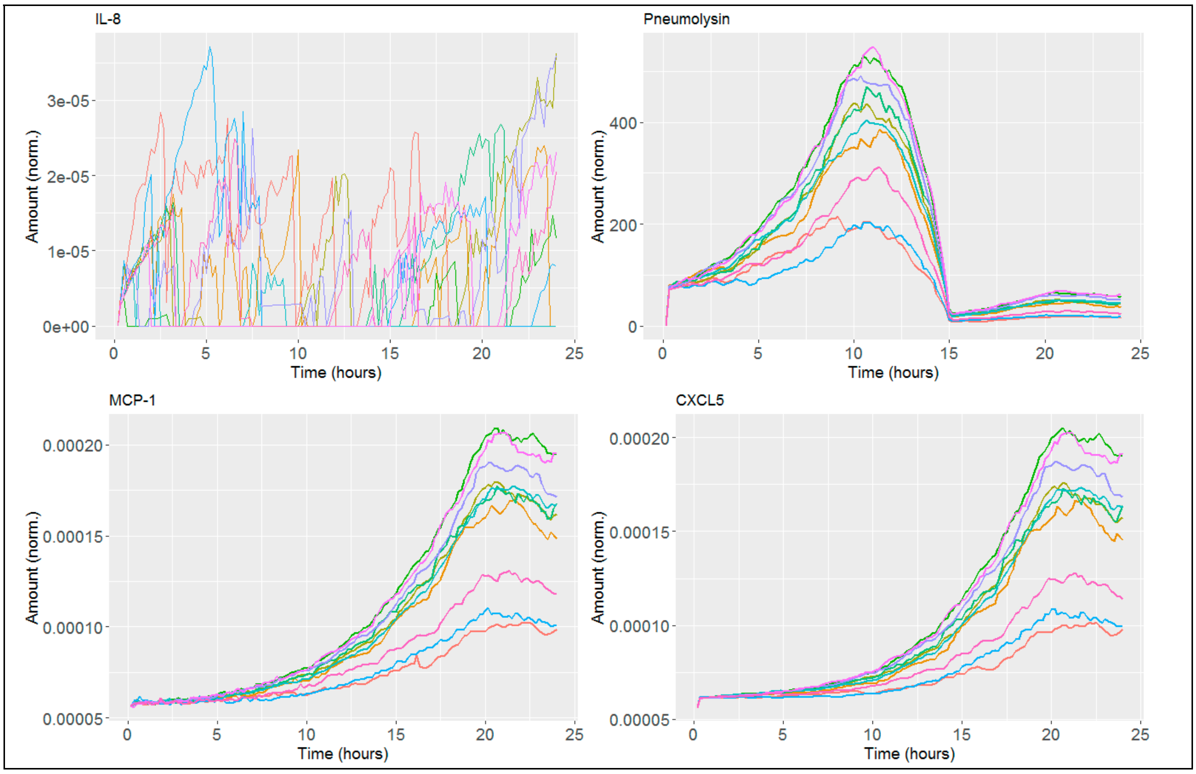

Figure S1. Diffusing variables of selected solutions from Figure 4.

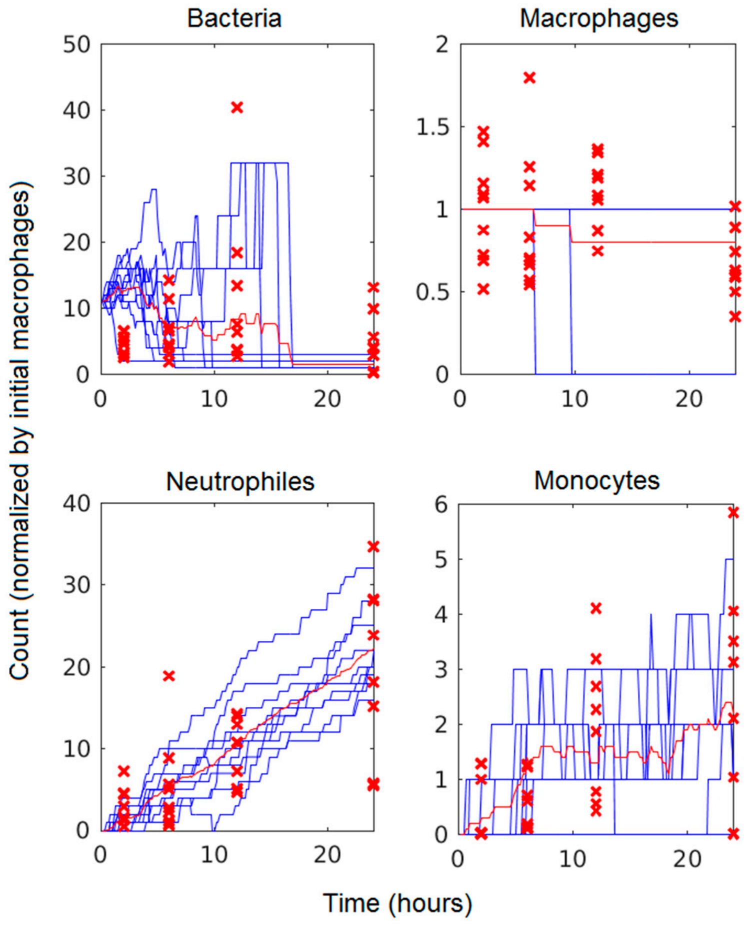

Figure S2. Parameter fitting with *in vivo* data. All the parameters labelled with "calibrated" in Table S1 were fitted to reproduce the experimental data from Berger et al., 2018[25]. Y axis unit are counts normalized by the initial macrophage number, and X axis unit accounts for hours.

Table S1. Parameter values of nominal solution of the model.

**Name**	**Value**	**Source**
Alveolar radius	100 µm	Ochs et ali, 2004[28]
Lining liquid width	1 µm	Lindert et al., 2007[29]
Diffusion constants of chemokines	Estimated from their molecular weight	Tang et al., 2016[30]
Lining liquid flow rate	$4.2 \cdot 10^{-2}$ min^{-1}	Lindert et al., 2007[29]
S. pneumoniae doubling time	200 min	Jakubovics, 2008[31]

S. pneumoniae doubling diameter (without capsule)	0.5 µm	Todar, 2003[32]
S. pneumoniae doubling diameter (with capsule)	0.75 µm	Todar, 2003[32]
Time to change pneumolysin production after infection	15 hours	Feldman et al., 1990[33]
Probability of surviving in presence of penicillin during 24 hours	0.0035	Tateda et al., 1996[26]
Number of macrophages at resting	Normalized to 1	Wallace et al., 1992[34]
Macrophage diameter	21 µm	Krombach et al., 1997[35]
Monocyte half life	6.62 hours	Doherty et al., 1988[36]
Movement velocity of phagocytes in the alveolus	2 µm/min	Khang, 2015[37]
Phagocytosis rate	0.048 min^{-1}	Athamna and Ofek, 1988[38]
Number of bacteria to produce macrophages apoptosis	87	Srivastava et al., 2005[39]
Time of exposition to pneumolysin to trigger apoptosis in macrophages	105 min	González-Juarbe[40]
Kinetic constant of monocyte recruitment	87 min^{-1}	Calibrated
Chemokine threshold to recruit monocytes	97 (arbitrary units)	Calibrated
Maximal monocyte recruitment rate	6 cells/min	Calibrated
Kinetic constant of neutrophil recruitment	212.88 min^{-1}	Calibrated
Chemokine threshold to recruit neutrophils	0.1944 (arbitrary units)	Calibrated
Maximal neutrophil recruitment rate	7.5809 cells/min	Calibrated
Neutrophil half life	2.6285 hours	Calibrated

Capsule production half time	19.81 hours	Calibrated
